# Retraction Note: High-mobility group box chromosomal protein-1 deletion alleviates osteoporosis in OVX rat model via suppressing the osteoclastogenesis and inflammation

**DOI:** 10.1186/s13018-025-05623-4

**Published:** 2025-03-04

**Authors:** Haotao Yu, Wei Zhou, Zhihong Zhong, Ruixin Qiu, Guoquan Chen, Ping Zhang

**Affiliations:** Department of Orthopedics, The Third Affiliated Hospital of Guangzhou Medical University, 63 Duobao Road, Liwan District, Guangzhou, Guangdong 510000 China

**Retraction Note: Journal of Orthopaedic Surgery and Research (2022) 17:232**

10.1186/s13018-022-03110-8

The Editor-in-Chief has retracted this article after concerns were raised about the data reported. Specifically:

- The β-actin blot in Fig. 2d appears similar to an image included in Fig. 1k of a prior publication [1], with no common authors.
- The HE panel of WT OVX in Fig. 3a appears similar to an image included in Fig. 5b of a prior publication [2], with no common authors.
- The COI1A1 and DLX2 blots in Fig. 5i appear similar to images included in Fig. 4f of a separate publication with no common authors [3], which was published around the same time as this publication.

The authors were not able to provide raw data for this publication upon request by the Publisher. The Editor-in-Chief no longer has confidence in the findings and conclusions of this article.

The authors did not respond to correspondence from the Publisher regarding this retraction.
